# Modeling the energetic cost of cancer as a result of altered energy metabolism: implications for cachexia

**DOI:** 10.1186/s12976-015-0015-0

**Published:** 2015-09-15

**Authors:** Douglas E. Friesen, Vickie E. Baracos, Jack A. Tuszynski

**Affiliations:** Department of Oncology, University of Alberta, Edmonton, AB T6G 1Z2 Canada; Department of Physics, University of Alberta, Edmonton, AB T6G 2E1 Canada

## Abstract

**Background:**

Cachexia affects most patients with incurable cancer. We hypothesize that in metastatic cancer the mass of the tumor as well as its level of anaerobic energy metabolism play a critical role in describing its energetic cost, which results in elevated resting energy expenditure and glucose utilization, leading to cachexia. Prior models of cancer cachexia may have underestimated the specific energetic cost of cancer as they have not taken the range of tumor mass and anaerobic energy metabolism fully into account.

**Methods:**

We therefore modelled the energetic cost of cancer as a function of the percentage of energy the cancer produces anaerobically, based on resting energy expenditure, glucose turnover, glucose recycling, and oxygen consumption in cancer patients found in previous studies.

**Results:**

Data from two clinical studies where tumor burden was estimated and resting energy expenditure or oxygen consumption were measured lead to a broad range of estimates of tumor cost from 190 to 470 kcal/kg tumor/day. These values will vary based of the percentage of energy the cancer produces anaerobically (from 0 to 100 %), which in and of itself can alter the cost over a 2 to 3-fold range. In addition to the tumor cost/kg and the degree of anaerobic metabolism, the impact on a given individual patient will depend on tumor burden, which can exceed 1 kg in advanced metastatic disease. Considering these dimensions of tumor cost we are able to produce a 2-dimensional map of potential values, with an overall range of 100–1400 kcal/day.

**Conclusions:**

Quantifying the energetic cost of cancer may benefit an understanding of the tumor’s causation of cachexia. Our estimates of the range of tumor cost include values that are higher than prior estimates and suggest that in metastatic disease the tumor cost could be expected to eclipse attempts to stabilize energy balance through nutrition support or by drug therapies. Tumor mass and the percentage of anaerobic metabolism in the tumor contribute to the cost of the tumor on the body and potentially lead directly to negative energy balance and increased muscle wasting.

**Electronic supplementary material:**

The online version of this article (doi:10.1186/s12976-015-0015-0) contains supplementary material, which is available to authorized users.

## Background

Cancer cachexia affects over 1.3 million people in the United States annually [1]. It is associated with severe muscle wasting and reduced survival that cannot be fully reversed by nutritional support [2]. The causes of cachexia are complex and not well understood [3], although its consequences are well documented. Cachexia is associated with reduced caloric intake, inflammation, metabolic change, and fatigue [4]. It affects the majority of late stage cancer patients [5]. Cachexia results from a variable combination of decreased food intake and altered metabolism. This reduction in food intake can arise from primary anorexia as well as symptoms arising from the tumor or side effects from cancer treatment [6], although reduced food intake does not completely explain the weight loss seen in cachexic patients [7]. In attempting to find the primary cause of cancer cachexia, it has been suggested that cancer induces abnormalities in lipid, carbohydrate, and protein metabolism, reduces the efficiency of energy metabolism, and this elevates resting energy expenditure (REE), which may be a major determinant in patients developing cachexia [8]. Our paper builds upon the investigation of the contribution of cancer on REE by investigating in greater depth the energy usage and substrate usage of tumors in order to quantify the energy cost of cancer to the patient, to develop a better understanding of the cause of cancer cachexia from an energetic perspective. The challenge in arriving at a cost estimate of cancer is that while in many studies the resting energy expenditure (REE) of cancer patients is measured [8–10], uncoupling the energetic usage of the body and that of the cancer is difficult. If the cancer is dispersed at several locations its entire volume or mass is difficult to quantify, and the measurement of the specific metabolic rate (i.e. energy cost/kg of tissue) of a tumor mass *in vivo* is technically challenging in human subjects [11].

**Fig. 1:**
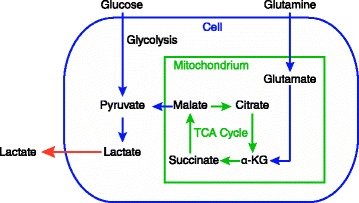
Metabolic pathways that convert glucose and glutamine into lactate. Glucose is converted via glycolysis to lactate, and glutamine through a truncated TCA cycle is able to be converted to lactate as well via glutaminolysis. α-KG stands for alpha-ketoglutarate

Tumors generally have a high uptake of glucose relative to most normal tissues, and this is exploited clinically in the use of ^18^ F-deoxyglucose positron emission tomography (FDG-PET) to detect cancer [12]. This upregulated glycolysis in cancer cells is a hallmark of cancer [13]. The high demand for glucose, even in the presence of adequate oxygen, has been termed the Warburg effect. To what extent a tumor generates ATP based on the glycolytic pathway converting glucose to pyruvate and then to lactate (an anaerobic process) versus oxidative phosphorylation (an aerobic process) is difficult to ascertain *in vivo* and likely varies considerably [11, 14]. In this paper, we refer to glycolysis as the pathway that converts glucose to lactate, generating 2 net ATP for the cell (Fig. 1). Warburg estimated that highly glycolytic tumors may make as much as 50 % of their ATP from glycolysis [15], although other researchers have found wide ranges of values *in vitro* (0.31 to 80 %) [16, 17]. *In vitro* studies have limitations, as cells may have increased glycolysis due to the artificial environment conducive to proliferation [18]. More relevant are recent studies examining the energy metabolism of tumors *in situ*, obtained by infusing uniformly labeled ^13^C-glucose into cancer patients and then performing surgical resection of the tumor followed by ^13^C nuclear magnetic resonance spectroscopy [11, 19, 20]. In brain tumors glycolysis was activated, though oxidative phosphorylation (oxphos) was still intact [19]. In lung tumors glycolysis was elevated compared to surrounding non-cancerous tissues [20]. Cancer patients exhibit increased whole body glucose turnover [21–24], increased Cori cycle activity, where lactate is recycled to glucose [21–23, 25], and increased gluconeogenesis [22, 25], suggesting that *in vivo* glycolysis is elevated in many tumors. Additionally, microcalorimetric measurements of isolated tumor and non-tumor tissue samples removed from humans showed tumors having a higher metabolic rate, with increasing malignancy correlated with a higher metabolic rate [26].

Glutamine is also converted into lactate in cancer cells *in vitro*, and in glioblastoma cells it was found that ~60 % of glutamine was metabolized through glutaminolysis to lactate [27] (Fig. 1). Anaerobic metabolism of glucose and glutamine in the tumor is potentially a direct driver of muscle protein catabolism, as muscle is the major metabolic source of carbon for gluconeogenesis and glutamine biosynthesis.

The clinical approach to abnormalities of human body weight is framed in the concept of energy balance. A discrepancy between energy intake and energy expenditure results in cancer-associated weight loss, and to stop this (i.e. achieve weight maintenance) or to reverse it and achieve weight gain, requires a quantitative understanding of both the energy costs of the body and those of the tumor. While it might be important to know if total tumor cost was likely to be 10, 100 or 1000 kcal/day, we have no clearly defined theoretical framework to determine this cost and therefore no clear clinical guideline of how much energy intake is required to achieve the desired body weight goals. We therefore propose a quantitative theoretical model to estimate the energetic cost of a tumor *in situ* based on the percentage of energy generated by the tumor anaerobically. We estimate the energetic cost of cancer based on resting energy expenditure (REE), glucose turnover, glucose recycling, and oxygen consumption in cancer patients. REE is assessed by indirect calorimetry, which measures oxygen consumption, carbon dioxide production, and urea excretion to derive the energy usage of the body [28]. This analysis can help explain how tumors directly impact elevated REE seen in cancer patients [10], which may lead to cancer cachexia.

### The model

#### Mathematical model of tumor cost based on tumor energy metabolism

**Fig. 2:**
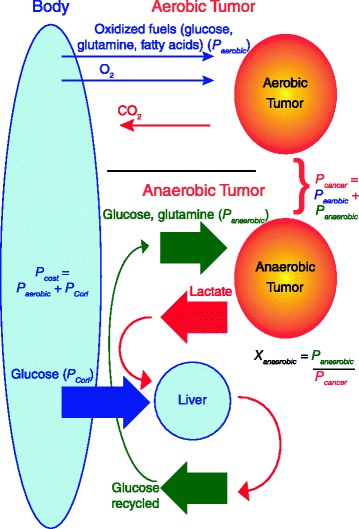
The cost of aerobic and anaerobic metabolism of the tumor on the body. A tumor will consume substrates both aerobically and anaerobically. The substrate usage of both an aerobic and an anaerobic tumor is described. Anaerobic metabolism costs the body three times more energy due to the cost of recycling lactate back into the glucose lost to the tumor (see Additional file 1 and equation (6)). The substrate usage is equivalent to describe the metabolic rates of the aerobic and anaerobic tumor, which leads to the definitions of the metabolic cost of the cancer on the body, *P*_*cost*_, and the metabolic rate of the tumor, *P*_*cancer*_. The percentage of energy produced anaerobically by the tumor, *X*_*anaerobic*_, is also illustrated

In order to quantify a possible cost of cancer based on the energy metabolism of tumors in patients, we formulate a model of the energetic cost of cancer based on its level of anaerobic energy production. Confusion has reigned on how to quantify the energetic cost of cancer in cachexic patients due to the complexity of correctly accounting for the recycling of glucose when it is converted to lactate by the tumor and then recycled primarily in the liver [29, 30]. We attempt to clarify this with our model, illustrated in Fig. 2. A cancer patient may be considered a system comprising the host and tumor mass. *P* denotes metabolic rate in kcal/day and *K* denotes the corresponding specific metabolic rate, in kcal/day per kg patient, tumor or other specified mass. The cancer’s energetic demand and growth will induce a cost on the normal body, and thus,

1 $$ P={P}_{normal}+{P}_{cost} $$

where *P*_*normal*_ is the metabolic rate of the person free of cancer, and *P*_*cost*_ is the energetic cost (in kcal/day) on the normal body caused by the cancer. *P*_*cost*_ is due to the metabolic requirements of the tumor, an elevated Cori cycle with increased gluconeogenesis, an activated immune system, an acute phase response, and increased substrate turnover [31–33]. In this paper, we investigate in detail the energetic cost of the tumor by focusing on the metabolic requirements of the tumor and the resultant elevated Cori cycle (Fig. 2). Normally, metabolic rates are determined based on oxygen consumption, and it is assumed that all food (protein, carbohydrates, and fat) is completely oxidized when the body is at rest. However, this is not the case for a tumor. We model a tumor having an anaerobic component of its energy metabolism. Thus:

2 $$ {P}_{cancer}={P}_{aerobic}+{P}_{anaerobic} $$

where *P*_*aerobic*_ is the aerobic component of a tumor’s metabolic rate, and *P*_*anaerobic*_ is the anaerobic component of a tumor’s metabolic rate (Fig. 2). Now, we introduce:

3 $$ {X}_{anaerobic}={P}_{anaerobic}/{P}_{cancer} $$

where *X*_*anaerobic*_ is the percentage of ATP energy generated anaerobically by the tumor cell. *X*_*anaerobic*_ is a measure of how anaerobic the tumor is, and will be used extensively in the analysis of how a tumor with a higher level of anaerobic metabolism will cost the body more energy.

While theoretically aerobic metabolism generates 38 ATP per glucose, when accounting for energy loss in the respiratory chain, current estimates indicate around 30 ATP are produced per glucose in oxidative phosphorylation [34]. Thus aerobic metabolism generates 15 times the ATP that anaerobic metabolism generates per glucose (30 ATP vs. 2 ATP). When energy is generated anaerobically by the tumor via glycolysis, 2 net ATP are generated per glucose converted to lactate, and then 6 ATP are needed by the body to reconvert the resulting lactate to glucose.

In a tumor producing energy 100 % aerobically, glucose from the body would be converted into 30 ATP for use by the tumor, and CO_2_ would return to the host. Other oxidized fuels from the body (such as glutamine and fatty acids) would also be obtained from the bloodstream and these tumor-oxidized fuels would be lost to the body. Thus, the cost of the tumor on the body, *P*_*cost*_, would be *P*_*aerobic*_. However, a tumor that produces energy 100 % anaerobically from glucose and glutamine produces only lactate, which will be recycled back into glucose in the liver and kidneys by the body via the Cori cycle [30]. Thus, all the glucose consumed by the tumor would be recovered, at a cost of 6 ATP per glucose used by the tumor. If a tumor consumed only glucose, a 100 % anaerobic tumor would consume 15 times the glucose that a 100 % aerobic tumor would consume, but the glucose would be recycled in the Cori cycle. In the anaerobic case, the Cori cycle takes 6 ATP to generate 2 ATP for the tumor, and so the energetic cost of the tumor is 3 times greater in the anaerobic case (see Additional file 1 for a more detailed derivation) (Fig. 2). Thus:

4 $$ {P}_{Cori}=3{P}_{anaerobic} $$

*P*_*Cori*_ is the energetic cost of Cori cycling lactate back into glucose (in kcal/day).

The energetic cost of cancer increases linearly as the percentage of energy derived anaerobically by the tumor, *X*_*anaerobic*_, increases. This can be expressed as follows:

5 $$ {P}_{cost}={P}_{aerobic}+{P}_{Cori} $$

6 $$ ={P}_{cancer}\left(1+2{X}_{anaerobic}\right) $$

where *P*_*cost*_ is the metabolic cost of the cancer on the body due to the cancer’s metabolism and the resultant Cori cycling that occurs to recycle the lactate produced by the cancer. (see Additional file 1 for a complete derivation). *P*_*cost*_ can be rewritten as:

7 $$ {P}_{cost}={K}_{cancer}{M}_{cancer}\left(1+2{X}_{anaerobic}\right) $$

This gives the total energetic cost of cancer as a function of the specific metabolic rate of cancer (*K*_*cancer*_), the mass of cancer (*M*_*cancer*_), and the percentage of ATP generated by the tumor anaerobically (*X*_*anaerobic*_). We attempt to estimate a range of tumor specific metabolic rates (*K*_*cancer*_) from several previous studies using measurements of REE and glucose turnover and Cori cycling activity, with the understanding that *K*_*cancer*_ may vary greatly between patients and tumors due to tumor heterogeneity of the disease, and in various microenvironment conditions, which may change rapidly in terms of glucose and oxygen availability.

Measurements currently performed when evaluating REE by indirect calorimetry, *REE*_*IC*_, will determine the following:

8 $$ RE{E}_{IC}={P}_{normal}+{P}_{cost} $$

9 $$ ={P}_{normal}+{P}_{aerobic}+{P}_{Cori} $$

Measured REE reportedly increases with increasing tumor burden [32, 35], which will be used to estimate *P*_*cost*_. Cancer will tend to have effects on the body in terms of weight loss, energy intake, cytokine production and an immune response, which may cause some systems to consume less energy than normal, such as that for digestion and movement, and some systems like the immune system to consume more energy. This has led to conflicting results on whether cancer leads to increased REE or not [7, 10, 36]. These values are not incorporated into *P*_*cost*_ in this analysis, and further studies would need to be done to control for these variables.

## Results

### Estimates of energetic cost of cancer based on REE studies

**Fig. 3:**
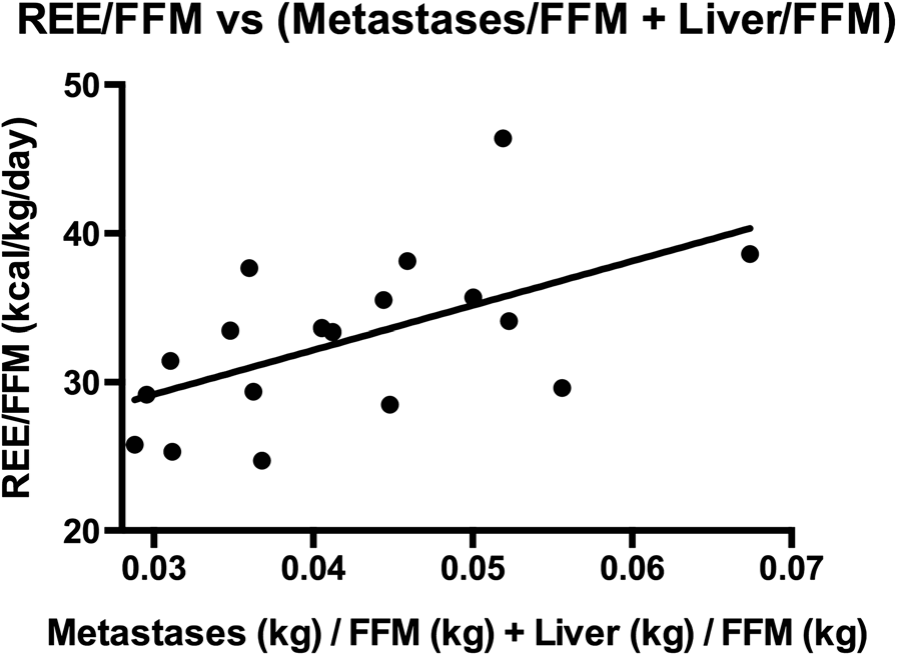
Impact of increased tumor mass on resting energy expenditure. Plot of resting energy expenditure (REE) and liver & metastases mass from the prospective colorectal cancer patient cohort (n = 18) found in [35]. The values have been adjusted for variations in fat free mass (FFM) of the patient cohort. Simple linear regression found: REE/FFM (kcal/kg/day) = (300 ± 110) * (Liver + Metastases mass) (kg)/FFM (kg) + 30 (r^2^ = 0.32, p = 0.01). Future studies to reduce the standard error of this result are recommended

The energetic cost of the tumor, *P*_*cost*_, can be estimated by the increase in *REE*_*IC*_ caused by the tumor. There are currently limited studies that concurrently measure *REE*_*IC*_ as well as estimate tumor burden. Study A [35] evaluated a metastatic colorectal cancer patient cohort (n = 18), with REE measurements and estimated mass of the combined liver and metastases located in the liver, determined by computed tomography image analysis. Patient fat-free mass (FFM) was measured as well. There is a primary correlation between REE and FFM, leading to the generalized prediction equation: REE = 370 + 21.6 × FFM (Cunningham equation) [37]. Healthy patients would also have liver mass primarily proportional to their fat free mass (FFM) [38], and so an increased (liver + metastases)/FFM value may be primarily due to increased cancer metastases. Thus we plotted the given patient REE compared to their estimated combined liver and metastases mass, adjusted for patient fat free mass (Fig. 3). We found:

10 $$ \begin{array}{l}\mathrm{R}\mathrm{E}\mathrm{E}\ \left(\mathrm{kcal}/\mathrm{day}\right)/\mathrm{F}\mathrm{F}\mathrm{M}\ \left(\mathrm{kg}\right)\ \mathrm{assessed}\ \mathrm{b}\mathrm{y}\ \mathrm{in}\mathrm{direct}\ \mathrm{calorimetry}\\ {}=300\pm 110\times \left(\mathrm{liver}+\mathrm{metastases}\kern0.5em \mathrm{mass},\kern0.5em \mathrm{in}\kern0.5em \mathrm{kg}\right)/\mathrm{F}\mathrm{F}\mathrm{M}\left(\mathrm{K}\mathrm{g}\right)+20\left({\mathrm{r}}^2=0.32,\mathrm{p}=0.01\right)\end{array} $$

It is important to note that there is a large standard error in this result. Ideally additional studies would be conducted to measure tumor mass and REE for patients over a time course in order to test more precisely how tumor mass might correlate with REE. As the liver’s metabolic rate is approximately 200 kcal/kg liver/day [39], a slope in Fig. 3 of 300 kcal/(kg liver + metastases)/day would potentially indicate the energetic cost of metastases is higher than 200 kcal/kg metastases/day. If we assume the increased REE after controlling for LBM is primarily due to metastases, then, as in our model, from equation (8) the specific total cost of the tumor from this study is (to 2 significant figures):

11 $$ {\mathrm{K}}_{cost}=300\pm 110\kern0.5em \mathrm{kcal}/\mathrm{kg}\kern0.5em \mathrm{tumor}/\mathrm{day}\kern0.5em \left(\mathrm{Study}\kern0.5em \mathrm{A}\right) $$

Again, this value must be used with caution due to the large standard error and imprecision in measuring tumor mass. However, we use this result to illustrate how our model may estimate the specific metabolic rate of the cancer using this slope of 300 kcal/kg tumor/day (Fig. 3), provided we know the percentage of ATP generated by glycolysis from the tumor. We make the initial assumption that 25 % of ATP is generated by glycolysis (*X*_*anaerobic*_ =0.25), as this is in line with *in vitro* estimates from cell lines derived from metastatic adenocarcinoma of the colon [40] and other cell lines where many estimates range from 20 to 75 % [41–43]. A value of 25 % anaerobic ATP synthesis is also consistent with preliminary *in situ* reports and *in vivo* data on glucose turnover and Cori cycling [23]. This corresponds to 50 % of the tumor-associated REE, *K*_*cost*_, due to Cori cycling and 50 % due to the aerobic component of tumor energy metabolism, if we neglect other factors that might increase REE as a result of the tumor (Additional file 2: Table S1 displays the relative *P*_*aerobic*_ and *P*_*Cori*_ based on *X*_*anaerobic*_). Thus:

12 $$ {K}_{aerobic}=150\pm 55\ \mathrm{kcal}/\mathrm{kg}\ \mathrm{tumor}/\mathrm{day} $$

13 $$ {K}_{Cori}=150\pm 55\ \mathrm{kcal}/\mathrm{kg}\ \mathrm{tumor}/\mathrm{day} $$

From this we obtain:

14 $$ {K}_{anaerobic}=50\pm 18\ \mathrm{kcal}/\mathrm{kg}\ \mathrm{tumor}/\mathrm{day} $$

15 $$ {K}_{cancer}=200\pm 73\ \mathrm{kcal}/\mathrm{kg}\ \mathrm{tumor}/\mathrm{day} $$

As these results are calculated scalar multiples of *K*_*cost*_ (see Additional file 2), the standard error in these values are multiples of the standard error of *K*_*cost*_. This value of *K*_*cancer*_ is higher than the previous assumption used by Hall of *K*_*cancer*_ = 150 kcal/kg/day [31], and is roughly equal to the metabolic rate of liver (Table 1) [39].

**Table 1:** Estimates of the energetic costs of cancer and comparable tissues

Organ	*K*_*aerobic*_ (kcal/kg/day)	*K*_*anaerobic*_ (kcal/kg/day)	*K*_*cancer*_ *or K*_*organ*_ (kcal/kg/day)	*K*_*Cori*_ (kcal/kg/day)	*K*_*cost*_ (kcal/kg/day)
Cancer: Study A [35]	150 ± 55	50 ± 18	200 ± 73	150 ± 55	300 ± 110
Cancer: Study B [32]	200 to 230	50 to 80	270 to 310	200 to 230	400 to 470
Liver [39]	200	0	200	0	200
Heart [39]	440	0	440	0	440
Kidney [39]	440	0	440	0	440
Brain [39]	240	0	240	0	240
Skeletal muscle [39]	13	0	13	0	13

The specific energetic cost of cancer, with *X*_*anaerobic*_ = 25 %, are compared with various other organs. Typical organs are assumed to have complete oxidation. Cost is rounded to two significant figures. Study A was a study of n = 18 metastatic colorectal cancer patients [35]. Study B was a study of n = 85 cancer patients studied preoperatively, with a majority of patients having solid tumors of the gastrointestinal tract, retroperitoneum, or limbs [32]. Ranges of cost were given due to patient mass in the studies not being provided. Details of calculations involved in *K*_*cost*_ for Study B are detailed in Additional file 3, and were based on the plot of oxygen consumption of patients and their tumor bulk with r^2^ = 0.79. Estimates of energetic costs of cancer should be taken with caution, as they could be highly variable due to the type of cancer studied

Another study related tumor mass with whole body oxygen consumption over a wide variety of types of cancers (Study B) [32]. Tumor mass was assessed by reviewing dimensions of tumors in resected specimens, as well as estimating volumes from ultrasound and computed tomographic scanning. Oxygen consumption was measured by indirect calorimetry. Their data corresponds to an oxidative metabolic increase of 6.67 kcal/kg tumor/day/kg patient, with r^2^ = 0.79 (see Additional file 3 for detailed calculations) [44]. Patient body mass data was not provided in Study B; however, assuming average patient weight between 60 and 70 kgs, the *K*_*cost*_ in Study B is estimated between 400 and 470 kcal/kg tumor/day (see Additional file 3). If again, ATP from glycolysis is estimated at 25 % for the tumor, this corresponds to *K*_*cancer*_ in the range of 270 to 310 kcal/kg/day (equation (6)). This estimate for specific metabolic rate of cancer falls within the range of previous estimates (150 to 406 kcal/kg/day [31, 35, 45]) (Table 1).

### Estimates of energetic cost of cancer based on substrate usage

**Fig. 4:**
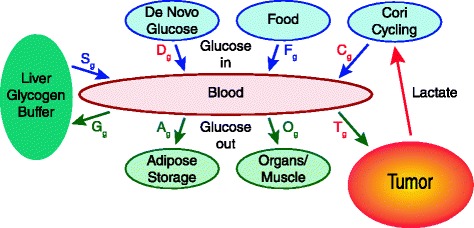
A model of glucose turnover in the bloodstream. The tumor, by consuming glucose at an elevated rate, *T*_*g*_, may lead to increased *de novo* glucose production, *D*_*g*_ (ie. muscle and fat catabolism), in fasting periods. This may be a large contributor to cachexia. As well, the Cori cycling rate, *C*_*g*_, a function of how much glucose is recycled in the liver from lactate generated from the body, leads to increased energy demand on the body when the tumor exports lactate due to its high rate of glycolysis. The liver glycogen buffer stores approximately 100-120 g of glucose

Another method to estimate specific tumor metabolic cost is to analyze glucose turnover in the body (i.e. rate of glucose entering and exiting the bloodstream) and the rate of Cori cycling (Fig. 4). Glucose is one of the primary sources of fuel for a tumor cell [43], and so estimating the glucose usage of the tumor will provide an estimate of a percentage of the total energy usage of the tumor. Glucose enters the bloodstream primarily from food (*F*_*g*_), glycogen stores (*S*_*g*_), de novo gluconeogenesis (*D*_*g*_), and Cori cycling (*C*_*g*_). Glucose is used by the body’s organs (*O*_*g*_), and may be stored as glycogen in the liver (*G*_*g*_), converted to triglyceride and stored in adipose tissue (*A*_*g*_), or used by a tumor (*T*_*g*_). The rate of all of these processes, at any given time, is

16 $$ {F}_g+{C}_g+{D}_g+{S}_g={O}_g+{T}_g+{A}_g+{G}_g $$

if we assume a static level of glucose in the blood. In a healthy person in the fed state, *F*_*g*_ will be high, *C*_*g*_, *D*_*g*_, and *S*_*g*_ will be essentially zero and storage (*G*_*g,*_*A*_*g*_) will occur. During early fasting, liver glycogen is mobilized to maintain blood glucose levels, and after a fast of 4–6 h, gluconeogenesis from the catabolism of muscle protein and glycerol from triglyceride will increasingly sustain blood glucose levels.

In the case of a patient with a tumor, *T*_*g*_ will be high continuously. Consider

17 $$ {T}_g={T}_{anaerobic}+{T}_{aerobic} $$

where *T*_*anaerobic*_ is the rate of glucose uptake by the tumor converted to lactate and *T*_*aerobic*_ is the rate of glucose that is used by the tumor to generate energy by oxidative phosphorylation. We will assume that *T*_*anaerobic*_ = *C*_*g*_ [46]. Then our rate equation becomes:

18 $$ {F}_g+{D}_g+{S}_g={O}_g+{T}_{aerobic}+{A}_g+{G}_g $$

*T*_*aerobic*_ will provide a constant drain on overall glucose supply, necessitate additional gluconeogenesis and correspondingly deplete gluconeogenic precursors, as *O*_*g*_, *A*_*g*_, and *G*_*g*_ will be reduced. All these factors may result in reduced liver glycogen stores, which have been reported in cachexic patients [22] and mice with cachexia-inducing C26 colon adenocarcinoma [47]. During fasting, *C*_*g*_ will supply some of the needed glucose, but as *F*_*g*_ is zero, and glycogen stores may be low, the tumor may increasingly rely on glucose originating from *de novo* gluconeogenesis, *D*_*g*_.

While a tumor has multiple fuels including glucose, glutamine, and fatty acids, if we analyze glucose turnover and glucose recycling, we can estimate the component of *P*_*cost*_ due to tumor glucose consumption, which we define as *P*_*cost_glucose*_ (see Additional file 4 for details on these calculations). Multiple studies gave measurements of glucose turnover and glucose recycling in cachexic patients, leading to our computation of estimates of *P*_*cost_glucose*_ in Table 2. Study C [23] compared patients with metastatic carcinoma who had progressive weight loss or were weight stable, and we calculate a *P*_*cost_glucose*_ = 200 kcal/day. Study D [22] compared cachexic metastatic colorectal cancer patients versus an age-related control group without cancer, with a calculated *P*_*cost_glucose*_ = 94 kcal/day. Study E [21] compared malnourished cachexic cancer patients versus malnourished patients without cancer, where all patients had lost ~14-15 % of their normal body weight, with a *P*_*cost_glucose*_ = 240 kcal/day.

**Table 2:** Cost estimates of tumors based on increased glucose turnover and increased glucose recycling

Study	Additional glucose turnover in cancer patients	*p*_*cancer*_ *,* probability glucose in bloodstream consumed by tumor	*X*_*anaerobic*_, % ATP generated from glycolysis in tumor	Cost estimate of tumor based on glucose turnover and glucose recycling (for 70 kg patient)
C [23]	2.06 g/kg patient/day	44 %	26 %	*P*_*cost_glucose*_ = 200 kcal/day
D [22]	0.850 g/kg patient/day	26 %	19 %	*P*_*cost_glucose*_ = 94 kcal/day
E [21]	1.19 g/kg patient/day	27 %	4 %	*P*_*cost_glucose*_ = 240 kcal/day

Additional information about the calculation of *p*_*cancer*_ *, X*_*anaerobic*_ *, K*_*cost*_, and *P*_*cost_glucose*_ are found in Additional file 4. Cost is rounded to two significant figures. Study C [23] compared patients with metastatic carcinoma who had progressive weight loss or were weight stable. Study D [22] compared cachexic metastatic colorectal cancer patients versus an age-related control group without cancer. Study E [21] compared malnourished cachexic cancer patients versus malnourished patients without cancer, where all patients had lost ~14-15 % of their normal body weight

The average of the studies C, D, and E gives an estimate of *P*_*cost_glucose*_ of 180 kcal/day for a 70 kg patient while fasting, although an estimate of the size of the tumors in these studies is not provided and so *K*_*cost_glucose*_ of the cancer cannot be calculated from these studies. Study B [32] related plasma glucose appearance to estimated mass of tumor in 85 cancer patients. From their data we calculate *K*_*cost_glucose*_ in the range of 220 to 260 kcal/kg tumor/day based on increased plasma glucose appearance dependent on tumor mass, and the assumption that 25 % of tumor ATP was generated anaerobically (see Additional file 4 for details on this calculation). This equates to *K*_*cost_glucose*_ being 55 % of *K*_*cost*_ in Study B.

### Tumor energetic cost

Table 3 summarizes estimates of the cost of the tumor on the body, *K*_*cost*_. The range of costs from REE and oxygen consumption is 190 to 470 kcal/kg tumor/day. This is a large range and further studies must incorporate estimates of tumor mass in order to derive more accurate values for *K*_*cost*_. However, analyzing this energetic cost, even with its current large uncertainty, is instructive in appreciating the potential importance of understanding this energetic cost of the tumor. Kleiber’s formula for estimating the reference man’s basal metabolic rate simplifies to [48]:

**Table 3:** Estimates of *K*_*cost*_ and *K*_*cancer*_

Study and parameter measured in study	Estimated cost of cancer, *K*_*cost*_ (kcal/kg/day)	Equivalent *K*_*cancer*_ assuming *X*_*anaerobic*_ = 25 % (kcal/kg/day)
A [35]: REE increase	300 ± 110	200 ± 73
B [32]: increased oxygen consumption	400 to 470	270 to 310

Estimates are summarized of tumor cost on body per kg tumor. The equivalent *K*_*cancer*_ using *X*_*anaerobic*_ = 25 % is shown (calculated from equation 6). Study A derives this estimate from increased resting energy expenditure (REE) per kg (liver + metastases) adjusted for variations in fat free mass (FFM) in a prospective colorectal cancer patient cohort (n = 18) found in [35], and Study B derives this estimate from increased oxygen consumption per kg tumor (see Additional file 3), in a study of n = 85 cancer patients studied preoperatively [32]. Ranges of cost in Study B were given due to patient mass in the studies not being provided. Cost is rounded to two significant figures. Estimates of energetic costs of cancer should be taken with caution, as they could be highly variable due to the type of cancer studied

19 $$ {P}_{normal}=69.6{M}^{0.75} $$

Thus, in our model, using the representative value of *K*_*cost*_ being 300 kcal/kg tumor/day from Study A, this leads to the metabolic rate of a cancer patient to be:

20 $$ P={P}_{normal}+{P}_{cost}=69.9{M}^{0.75}+300{M}_{cancer} $$

where *M* = *M*_*normal*_. We note the high coefficient based on tumor mass, and the fact that the cost scales linearly to the tumor mass.

**Fig. 5:**
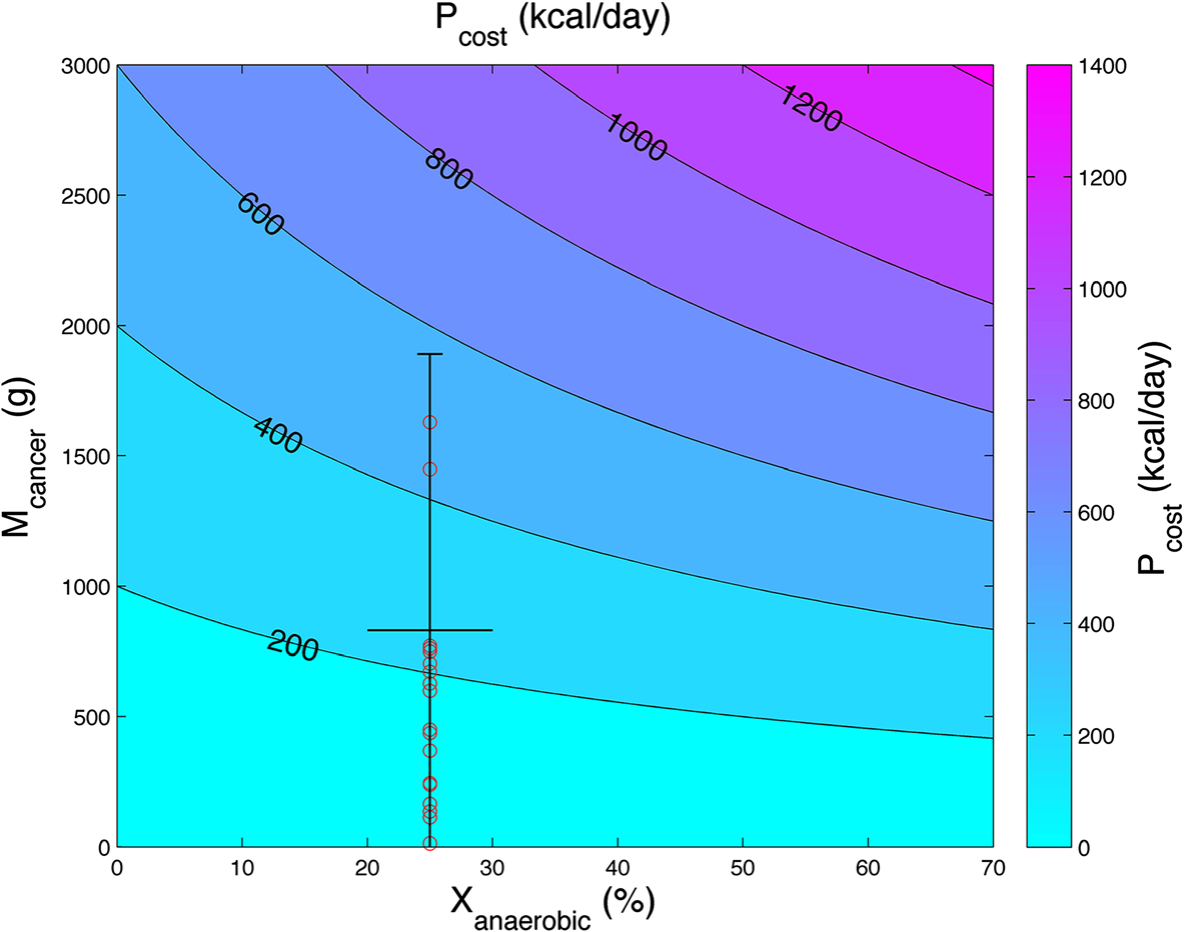
Contour plot of the estimated energetic tumor cost based on energy metabolism and tumor mass. The estimated energetic cost of the tumor, *P*_*cost*_, in kcal/day, is plotted based on its mass, *M*_*cancer*_, and the percentage of ATP the tumor generates anaerobically through glycolysis, *X*_*anaerobic*_. The plot uses equation (7), with *K*_*cancer*_ = 200 kcal/kg tumor/day found in Study A (see Table 3). Early stage tumors may not present a high cost, but as tumors grow and become more glycolytic, their cost will increase and may induce a catabolic, cachexic state. Patients from the retrospective colorectal cancer cohort in [35], n = 30 (Study A) are plotted in red, with assumed 25 % ATP generated by glycolysis, where tumor mass is estimated by taking their final (liver + metastases) mass and subtracting the initial (liver + metastases) mass in the final 12 months of their disease. This is to provide an illustration of where cachexic patients may fit within this map and should only be considered as a very rough estimate of tumor energetic cost. For the thirty patients, the mean tumor burden is 0.83 kg (equivalent to a cost of 250 kcal/day), standard deviation is 1.06 kg, and a patient with estimated tumor mass of 4.7 kg, outside the axis of the figure, is not shown

**Fig. 6:**
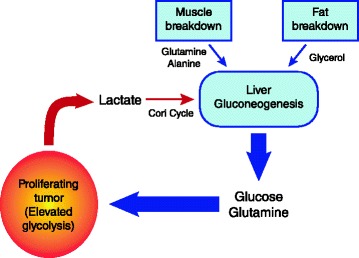
The proposed model of the tumor causing an energy deficit leading to cachexia. The tumor’s large consumption of glucose and glutamine, and conversion of these substrates to lactate which then require energy from the body to convert back to glucose, creates a vicious cycle of lost energy. Muscle breakdown would provide glucose and glutamine to feed the tumor, especially in a fasting state

To assess the range of values of the cost of the tumor, *P*_*cost*_, we plot equation (7),

$$ {P}_{cost}={K}_{cancer}{M}_{cancer}\left(1+2{X}_{anaerobic}\right) $$

using *K*_*cancer*_ = 200 kcal/kg tumor/day calculated from Study A, and a range of clinically plausible values of the mass of the cancer, *M*_*cancer*_, and the percentage of ATP generated anaerobically by the tumor, *X*_*anaerobic*_ (Fig. 5). Study A [35] provided an analysis of a retrospective colorectal cancer cohort (n = 30) of patients, tracking their liver + metastases mass over the final 12 months of their disease. As liver mass was assumed to be constant, we could provide an estimate of metastases mass at the endpoint of the disease, and these masses are plotted in red in Fig. 5, where we use the previous assumption that *X*_*anaerobic*_ = 0.25 for these patients. In early stage cancers, the cost of the tumor will likely be in the lower-left quadrant of Fig. 5 but for extensive metastatic disease, the tumor cost would extend towards the upper-right quadrant [32, 49, 50]. The potentially large energetic cost of the tumor may help explain cachexia in advanced metastatic disease. A disseminated metastatic tumor weighing 1.8 kg which makes 25 % of its ATP from glycolysis could plausibly cost ~540 kcal/day, i.e. 32 % of the basal metabolic rate of the reference man. Such high levels of tumor energy expenditure, often in the context of profound anorexia, would drive proteolysis and lipolysis. This model of cachexia is summarized in Fig. 6.

### Percentage of energy from glucose lost to the tumor

While a tumor may have a high energetic cost, its cost may not be readily apparent as measured by indirect calorimetry, because while a tumor might have a high energy usage, owing to depletion of lean and fat tissues the body may be correspondingly consuming less energy. By analyzing glucose turnover and Cori cycling we can estimate the percentage of energy from glucose lost to the tumor, which may be a parameter better suited to predict cachexia based on tumor energetic cost.

The probability that glucose is consumed by a tumor, *p*_*cancer*_*,* can be estimated as the increased glucose turnover in cancer patients compared to the glucose turnover of the patient:

21 $$ {p}_{cancer}=\left({t}_{cancer}-{t}_{control}\right)/{t}_{cancer} $$

where *t*_*cancer*_ is the glucose turnover rate in cancer patients and *t*_*control*_ is glucose turnover rate in healthy controls. *p*_*cancer*_ ranged from 26 to 44 % in studies C-E (Table 2). The estimate of the percentage of glucose converted to lactate by tumors, *p*_*anaerobic*_, can be estimated as the increased Cori cycling in cancer patients (indicating the amount of glucose used by the tumor anaerobically) compared to the increased glucose turnover (indicating the amount of glucose used by the tumor):

22 $$ {p}_{anaerobic}=\left({C}_{cancer}-{C}_{control}\right)/\left({t}_{cancer}-{t}_{control}\right) $$

where *C*_*cancer*_ is the Cori cycling rate of glucose in cancer patients and *C*_*control*_ is the Cori cycling rate of glucose in healthy controls. *p*_*anaerobic*_ ranged from 40 to 84 % in studies C-E (Table 2). These values of *p*_*anaerobic*_ correspond to 4 to 26 % of ATP generated from glycolysis (*X*_*anaerobic*_) (Table 2 and Additional file 4). A tumor has a much higher *p*_*anaerobic*_ than *X*_*anaerobic*_ as the ATP generated anaerobically per glucose is 15 times less than that of ATP generated aerobically from glucose (see Additional file 4 for the exact conversion formula).

**Fig. 7:**
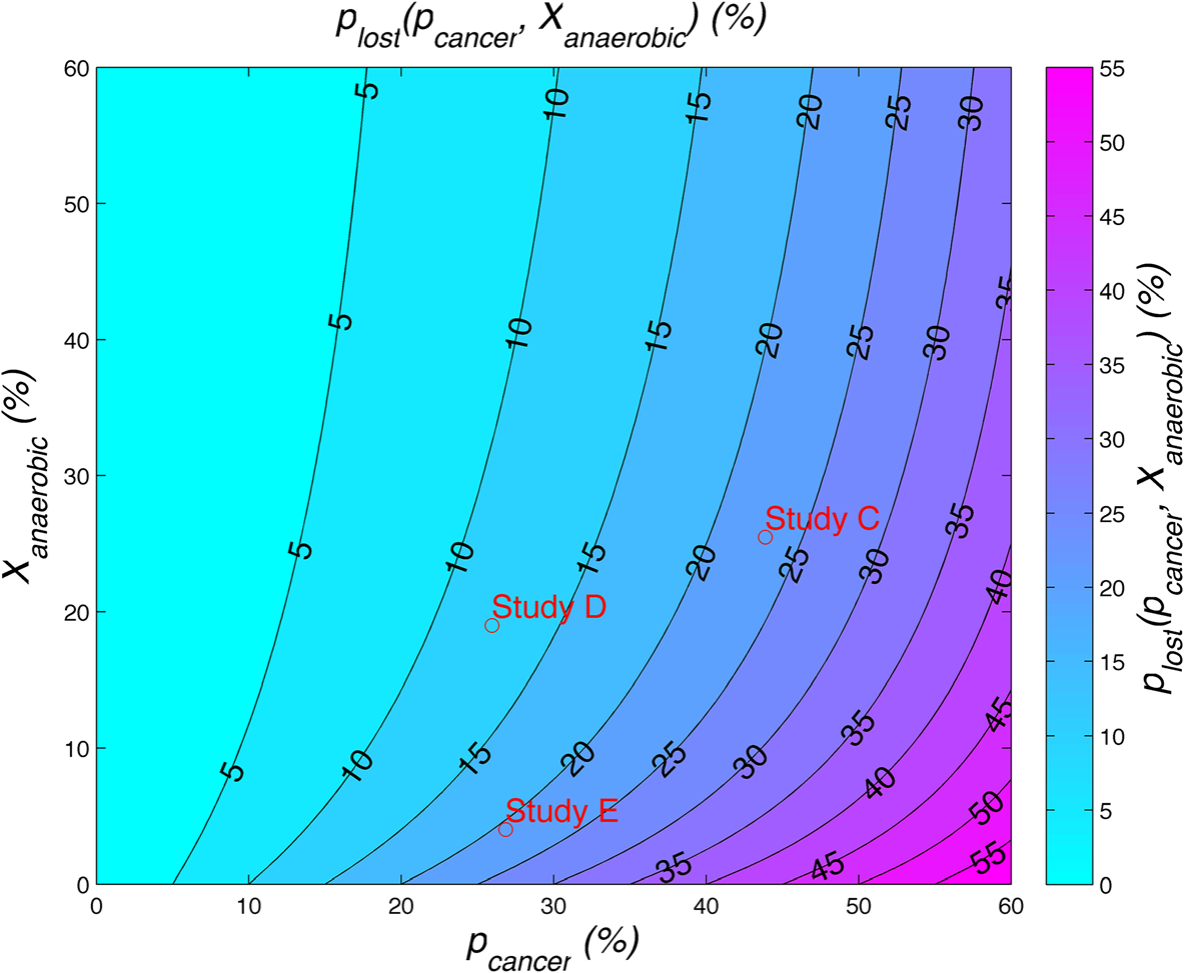
Contour plot of the percentage of glucose energy lost to tumor, *p*_*lost*_. *p*_*lost*_ is indicated on the graph for studies C-E based on the probability the glucose in the bloodstream enters the tumor, *p*_*cancer*_, and the percentage of ATP derived by glycolysis in the tumor, *X*_*anaerobic*_. Study C compared cachexic cancer patients versus weight-stable cancer patients. Studies D and E compared cachexic cancer patients versus normal controls. In study E both cachexic cancer patients and normal controls were 14-15 % under their normal weight

The expected ATP generated for the body per glucose entering the bloodstream (energetic payout of a glucose), *g(p*_*cancer*_*, p*_*anaerobic*_*)*, can be estimated by assuming that 30 ATP will be generated per glucose consumed by the body [34], and that 6 ATP will be consumed to recover a glucose used by tumor anaerobically, and 0 ATP will be generated if the glucose is lost to the tumor aerobically (see Additional file 5 for more information on the definition of *g(p*_*cancer*_*, p*_*anaerobic*_*)*). The percentage of energy lost to the body per glucose entering the bloodstream, *p*_*lost*_, is then determined from *g(p*_*cancer*_*, p*_*anaerobic*_*)* (see Additional file 5). *p*_*lost*_ is plotted in Fig. 7. The values of *p*_*cancer*_ and *X*_*anaerobic*_ are calculated for studies C-E in order to find *p*_*lost*_ for these studies (see Table 2). Study C compared cachexic cancer patients to weight-stable cancer patients and showed an additional 23 % of energy from glucose lost. Studies D and E, which compared cancer patients to normal controls showed 12 % and 21 % of energy from glucose lost to the tumor, respectively.

## Discussion

We used a variety of currently available evidence for REE, glucose turnover, Cori cycling rate, and tumor burden to obtain our main result to estimate a tumor’s energy cost on the body, *P*_*cost*_, based on tumor mass (*M*_*cancer*_), the percentage of ATP synthesized anaerobically in the tumor (*X*_*anaerobic*_), and the specific metabolic rate of the cancer (*K*_*cancer*_) (Fig. 5). The first dimension of the map (*M*_*cancer*_) encompasses a range of clinically plausible tumor burdens up to 3 kg [32, 51]; the second dimension is *X*_*anaerobic*_ over a range primarily seen in cancer cell lines [14–16], and we use a base value of *K*_*cancer*_ = 200 kcal/kg tumor/day estimated from Study A [35]. This map provides a range of estimates, which may be considered within the limitation that data sets which include all of the relevant parameters: *M*_*cancer*_, *X*_*anaerobic*_, and *K*_*cancer*_, with known REE values over the time course of the disease, are not readily available. In future studies, *M*_*cancer*_ and insight into glucose utilization could be aided by combined positron emission tomography/computed tomography (PET/CT) scan analysis [52]. *X*_*anaerobic*_ and *K*_*cancer*_ are difficult to evaluate empirically in a direct manner, with current efforts involving *in vivo* isotope labelling, primarily with ^13^C-glucose [11]. In human cancers, *X*_*anaerobic*_, could be lower or higher than the base value we used in our model (25 %), and could also vary over time and even within a tumor [11]. Within those caveats, estimates of *P*_*cost*_ are higher than previously considered [31, 35]. Consider a metastatic colon cancer patient with the average tumor burden of the sample in Fig. 5, for which the energetic cost of the tumor would likely fall in the range of 180–500 kcal/day, depending on the proportion of ATP synthesized anaerobically within that tumor mass. At the distal ends of the tumor mass distribution in the patient sample, there are individuals whose tumor cost would be < 200 kcal/day in any instance, and others whose tumor cost could be in excess of 400 kcal/day and potentially over 800 kcal/day if largely anaerobic. These estimates of tumor energy demand are useful in achieving understanding of the scope of potential tumor contribution to the body’s energetic deficit. The absolute cost of a tumor will have a variable impact on patients depending on their REE which is largely dependent on body mass. For instance, a tumor cost of 300 kcal/day will be 25 % of REE of a patient with a normal REE of 1200 kcal/day, but only 15 % of REE for a patient with a normal REE of 2000 kcal/day.

The estimate of % ATP synthesis generated anaerobically, *X*_*anaerobic*_, is a large assumption of our model, and further information on tumor metabolism *in situ* in humans is needed to refine this number for various cancers and at various stages of disease progression [11]. Drug-resistant, aggressive tumors found in late stage cancer patients may have a higher rate of glycolysis [26, 53]. A study to investigate energy consumption in the resting versus proliferating state, using mitogen-activated rat thymocytes, found cells in the proliferating state consumed 4.9 times the ATP as those in the resting state, with 86 % of ATP generated from glycolysis in the proliferating state versus only 12 % of ATP generated from glycolysis in the resting state [54]. Thus, rapidly proliferating tumors may have increased *X*_*anaerobic*_ and *K*_*cancer*_, which would drive *P*_*cost*_ higher according to equation (7). This is consistent with findings of elevated REE for newly diagnosed stage IV cancer patients compared with newly diagnosed stage I-III cancer patients [8].

Analyzing glucose turnover and glucose recycling also approximated the energetic cost of cancer where glucose is the energy substrate, *P*_*cost_glucose*_, in Table 2. These calculations, perhaps more importantly, allow us to approximate the percentage of energy taken from the body from glucose by the cancer. Approximately 10-25 % of energy derived from glucose is lost to the tumor in cachexic cancer patients (Fig. 7). This may lead to muscle wasting to generate more glucose to make up for this loss of energy. It also suggests a further avenue of study to test for a critical percentage of energy from glucose lost, *p*_*lost*_, which overloads the body’s ability to maintain adequate glucose to the body without resorting to excessive gluconeogenesis and muscle wasting. In effect, we hypothesize this parameter, *p*_*lost*_, may be a predictor for the onset of cachexia.

Our model develops further the previous model of Hall and Baracos [31] by refining estimates of the cost of the tumor because of the tumor’s increased glucose consumption, and incorporates the possibility that a tumor may vary in the proportion of oxidative and glycolytic metabolism. Hall *et al.* [31] modeled the change in lipolysis, proteolysis, gluconeogenesis and Cori cycle rates during progressive tumor growth and their effects on resting metabolic rate and gluconeogenesis. The model incorporated the cost of elevated glycogen, fat, and protein turnover and lipolysis and proteolysis. It also incorporated the cost of the tumor in terms of Cori cycling cost, which was estimated to start at 16 kcal/day and increased to 64 kcal/day, and assumed a specific metabolic cost of the tumor at 150 kcal/kg/day based on experimental studies. Our model refines this to a base estimate of *K*_*aerobic*_ = 150 kcal/kg/day, and *K*_*Cori*_ based on the level of glycolysis in the cancer, with a base estimate of 150 kcal/kg/day, for a combined total cost, *K*_*cost*_, of 300 kcal/kg/day (Study A). Based on this *K*_*cost*_, and assuming *X*_*anaerobic*_ is 25 %, the actual specific metabolic rate of cancer, *K*_*cancer*_, here is estimated at 200 kcal/kg/day.

### Implications of tumor anaerobic metabolism for skeletal muscle loss

Anaerobic metabolism may drive additional gluconeogenesis, due to the increased usage of glucose and glutamine. Cancer is suggested to act as a “glutamine trap,” leading to a transfer of nitrogen from muscle to the tumor [55]. Cultured tumor cells require ten times as much glutamine as any other amino acid [27] and more than 90 % of the body’s glutamine stores are in the muscle [56]. It is now recognized that glucose and glutamine are the main sources of energy for cancer cells [27, 43], although this has yet to be conclusively established *in vivo*. Since skeletal muscle–derived amino acids are the major precursors of glutamine synthesis as well as the main source of carbon for gluconeogenesis, muscle protein catabolism may be driven by tumor consumption of these substrates.

### Clinical implications

A dilemma in treating patients with cachexia is that an increase in caloric consumption reduces or slows weight loss but does not typically lead to weight gain [57, 58]. This raises the question as to exactly how much energy intake would be required to result in weight stability or restore positive energy balance. Improved volitional energy intake that is achieved with dietary consultation and oral nutritional supplements can reach between 500 and 600 kcal/day [59, 60]. This type of intervention has documented clinical benefits and is most successful during radiation and chemotherapy with curative intent, while the tumor is responding to treatment. Indications for non-volitional (artificial enteral/parenteral) feeding are specified within published clinical practice guidelines [61, 62], according to their potential benefits and risks. The reference range of tumor energy expenditure (Fig. 5) should help frame clinical expectations. For the patient with limited tumor burden or whose disease is controlled by anticancer therapy, reduced weight loss or weight stability could be achievable within a realizable set of nutritional goals. Patients undergoing an objective tumor response during treatment (tumor shrinkage) would be expected to have a reduced tumor energy demand compared to a rapidly proliferating tumor. Aligned with the concept of refractory cachexia [2] for the patient whose cancer is metastatic, very large and growing in spite of cancer therapy, the tumor cost would be expected to eclipse attempts to stabilize energy balance through volitional food intake, or even by means of artificial nutrition. Additionally, any proposed treatment for cachexia, such as reducing the activity of catabolic mediators (ie. cytokines, myostatin) that activate proteolysis and lipolysis, without addressing the energetic burden of the cancer will potentially have limited impact.

## Conclusions

We have calculated the energetic cost of cancer based on the cancer’s specific metabolic rate and level of anaerobic energy production, and estimated this cost based on clinical data, reaching the conclusion that tumor cost may be considerably higher than previously assumed in patients with metastatic disease. High glucose turnover as a result of anaerobic energy production has the potential to result in cachexia due the high constant demand for glucose from the tumor, especially in the fasting state. Our models in Figs. 2 and 4 provide a framework for better understanding the role of anaerobic energy production in cancer in affecting the energy balance in cancer patients. Our estimates of the energetic cost of tumors as a function of anaerobic energy production in the tumor in Fig. 5 and equation 7 suggest that reduction in anaerobic tumor ATP synthesis may mitigate tumor cost. At present we do not have a means of convincing a tumor to switch to aerobic metabolism, although this becomes a topic of interest now that we understand that such an intervention could have a quantitatively important impact on energy balance. While it is generally understood that hypermetabolism is common in advanced cancer patients [10, 63], future studies should attempt to estimate tumor burden, tumor energy consumption through indirect calorimetry, tumor substrate utilization, and ideally liver glycogen reserves at different stages of cancer disease progression in order to better understand the tumor’s energy consumption as a cause of hypermetabolism and weight loss.

## Additional files

Additional file 1:
**Tumor cost derivation.** Additional details of the derivation of equations 5 and 6 are presented. (PDF 69 kb) Additional file 2: Table S1. Percent of increased measured tumor energy expenditure, *P*_*cost*_ = *P*_*Cori*_ *+ P*_*aerobic*_, due to different metabolic pathways, according to our model, at various percentages of ATP supplied by glycolysis, *X*_*anaerobic*_. (PDF 58 kb) Additional file 3:
**Tumor cost from oxygen consumption increase.** Additional details of the calculation of tumor cost from oxygen consumption increase in Study B are given. (PDF 72 kb) Additional file 4:
**Calculations of cost of tumor based on glucose utilization.** Additional details are given on the calculations performed to obtain the values presented in Table 2. (PDF 71 kb) Additional file 5:
**Recurrence relation** ***g(p***_***cancer***_ ***, p***_***anaerobic***_ ***)***. Additional explanation is given to the recurrence relation *g*(p_*cancer*_ *, p*_*anaerobic*_ *)* and how it is used to calculate *p*_*lost*_. (PDF 70 kb)
