# Supplementary material for: Modeling the energetic cost of cancer as a result of altered energy metabolism: implications for cachexia
Source: Theor Biol Med Model. 2015 Sep 15;12:17. doi: 10.1186/s12976-015-0015-0 (PMC4570294; doi:10.1186/s12976-015-0015-0)
Supplement: Additional file 1: — Tumor cost derivation. Additional details of the derivation of equations 5 and 6 are presented. (PDF 69 kb) [file 12976_2015_15_MOESM1_ESM.pdf]

Additional File 1: Tumor cost derivation

The cost to the body of the tumor in terms of glucose is:

$$\textit{Glucose cost} = \textit{Glucose lost to aerobic portion of tumor} + \textit{glucose equivalents required to recycle lactate generated anaerobically by tumor} \quad (1)$$

This is because the glucose consumed anaerobically by the tumor is converted to lactate and is released back into the bloodstream. The glucose consumed aerobically by the tumor is not reusable by the body.

Consider a tumor consuming ATP at a rate of A_{cancer} (mol ATP/day), where

$$A_{cancer} = A_{aerobic} + A_{anaerobic} \quad (2)$$

Then,

$$Glucose_{cost} = A_{aerobic} /30 + A_{Cori}/30 \quad (3)$$

$$= A_{aerobic} /30 + 6(A_{anaerobic}/2)/30 \quad (4)$$

$$= (A_{aerobic} + 3\ A_{anaerobic}) / 30 \quad (5)$$

$$= (A_{cancer} + 2\ A_{anaerobic}) / 30 \quad (6)$$

$$= (A_{cancer}/30)(1+2X_{anaerobic}) \quad (7)$$

$Glucose_{cost}$ is the rate glucose is consumed as a direct result of the tumor, in (mol glucose/day). A_{Cori} is the rate of ATP consumed by the Cori cycle to reconvert the lactate to glucose. The first term in equation (3) is the glucose used by the aerobic portion of the tumor, and the second term refers to the glucose needed to generate the ATP in the liver to convert the lactate generated by the anaerobic portion of the tumor, using the fact that a glucose yields 30 ATP [1]. Equation (4) uses the fact that 6 ATP are required to convert lactate back to glucose, and $A_{anaerobic}/2$ is the amount of glucose used anaerobically by the tumor, as glycolysis in a cell generates 2 ATP. Equation (6) uses

$$A_{cancer} = A_{aerobic} + A_{anaerobic} \quad (8)$$

Equation (7) uses

$$X_{anaerobic} = A_{anaerobic} / A_{cancer} \quad (9)$$

The aerobic component of the tumor metabolism includes glucose and all other fuels consumed aerobically.
If $X_{anaerobic}$=1, we see from equation (7) that the glucose cost is three times as much as if $X_{anaerobic}$=0. Thus, we say that an anaerobic tumor costs three times as much as an aerobic tumor. This is graphically illustrated in Figure 2.

As ATP is the currency of energy in the cell, equation (7) can be rewritten as:

$$P_{cost} = P_{cancer}(1+2\ X_{anaerobic}) \quad (10)$$

which is equation (5-6) in the main text.

References

1. Rich PR: **The molecular machinery of Keilin's respiratory chain.** *Biochem Soc Trans* 2003, **31:**1095-1105.
