# Supplementary material for: Modeling the energetic cost of cancer as a result of altered energy metabolism: implications for cachexia
Source: Theor Biol Med Model. 2015 Sep 15;12:17. doi: 10.1186/s12976-015-0015-0 (PMC4570294; doi:10.1186/s12976-015-0015-0)
Supplement: Additional file 2: Table S1. — Percent of increased measured tumor energy expenditure, P cost = P Cori + P aerobic, due to different metabolic pathways, according to our model, at various percentages of ATP supplied by glycolysis, X anaerobic. (PDF 58 kb) [file 12976_2015_15_MOESM2_ESM.pdf]

Additional File 2: Table S1

Table S1:

Percentage glycolysis $X_{anaerobic}$ (%)	% of P_{cost} due to Cori cycle (P_{Cori}/P_{cost}) (%)	% of P_{cost} due to aerobic portion of tumor ($P_{aerobic}/P_{cost}$) (%)
0	0	100
5	14	86
10	25	75
20	43	57
25	50	50
50	75	25
60	82	18
85	94	6
100	100	0

Table S1. Percent of increased measured tumor energy expenditure, $P_{cost} = P_{Cori} + P_{aerobic}$, due to different metabolic pathways, according to our model, at various percentages of ATP supplied by glycolysis, $X_{anaerobic}$.

In effect, the anaerobic tumor due to the Cori cycle costs 3 times an aerobic tumor (see Additional file 1).

So:

$$P_{Cori} / P_{aerobic} = 3\ X_{anaerobic} / (1 - X_{anaerobic})) \qquad (1)$$

As in equation (5) in the main text:

$$P_{cost} = P_{aerobic} + P_{Cori} \qquad (2)$$

So:

$$P_{Cori}/P_{cost} = 3\ X_{anaerobic} / (3\ X_{anaerobic} + (1 - X_{anaerobic})) \qquad (3)$$

$$= 3\ X_{anaerobic} / (2\ X_{anaerobic} + 1) \qquad (4)$$

Likewise:

$$P_{aerobic}/P_{cost} = (1 - X_{anaerobic}) / (3\ X_{anaerobic} + (1 - X_{anaerobic})) \qquad (5)$$

$$= (1 - X_{anaerobic}) / (2\ X_{anaerobic} + 1) \qquad (6)$$

These values are shown in Table S1 in columns 2 and 3.
