# Supplementary material for: Modeling the energetic cost of cancer as a result of altered energy metabolism: implications for cachexia
Source: Theor Biol Med Model. 2015 Sep 15;12:17. doi: 10.1186/s12976-015-0015-0 (PMC4570294; doi:10.1186/s12976-015-0015-0)
Supplement: Additional file 3: — Tumor cost from oxygen consumption increase. Additional details of the calculation of tumor cost from oxygen consumption increase in Study B are given. (PDF 72 kb) [file 12976_2015_15_MOESM3_ESM.pdf]

Additional file 3: Tumor cost from oxygen consumption increase

In Koea and Shaw [1], oxygen consumption (µmol / kg min) as a function of tumor bulk (g) is given. The slope is 41 µmol / kg tumor/ min/kg patient (r^2=0.79). To convert this into the cost of cancer as a function of tumor bulk in kcal/kg tumor/day/kg patient, the oxygen consumption per tumor bulk (µmol / kg tumor/ min/kg patient) is converted to ml oxygen/kg tumor/min/kg patient) using the conversion 0.022391 ml = µmol O_2. This is converted to kJ/kg tumor/min/kg patient by the conversion 21.1 kJ/ O_2 [2]. This is converted to kcal/kg tumor/day/kg patient using standard conversions. As patient mass was not given in this study, converting this value precisely to kcal/kg tumor/day was not possible; however we assume average patient weight was between 60 and 70 kg [3] to offer a range of values of tumor cost in kcal/kg tumor/day.

References

1. Koea JB, Shaw JH: **The effect of tumor bulk on the metabolic response to cancer.** *Ann Surg* 1992, **215:**282-288.
2. Scott CB: **Contribution of anaerobic energy expenditure to whole body thermogenesis.** *Nutr Metab* 2005, **2:**14.
3. Xu WP, Cao DX, Lin ZM, Wu GH, Chen L, Zhang JP, Zhang B, Yang ZA, Jiang Y, Han YS, et al: **Analysis of energy utilization and body composition in kidney, bladder, and adrenal cancer patients.** *Urologic oncology* 2012, **30:**711-718.
