# Supplementary material for: Modeling the energetic cost of cancer as a result of altered energy metabolism: implications for cachexia
Source: Theor Biol Med Model. 2015 Sep 15;12:17. doi: 10.1186/s12976-015-0015-0 (PMC4570294; doi:10.1186/s12976-015-0015-0)
Supplement: Additional file 4: — Calculations of cost of tumor based on glucose utilization. Additional details are given on the calculations performed to obtain the values presented in Table 2. (PDF 71 kb) [file 12976_2015_15_MOESM4_ESM.pdf]

Additional file 4: Calculations of cost of tumor based on glucose utilization

p_{cancer} is calculated using equation (21) in the main text.

To calculate the estimated cost of the tumor based on glucose turnover and glucose recycling, $P_{cost\_glucose}$, equations (22) in the main text are used to calculate $p_{anaerobic}$. This assumes that the increased glucose turnover enters the cancer, and that the increased Cori cycling is due to glucose being recycled as a result of the glucose being metabolized anaerobically by the tumor. $p_{anaerobic}$ is equivalent to $X_{anaerobic}$ using the following formula (assuming glucose is used for either aerobic or anaerobic energy)

$$X_{anaerobic} = p_{anaerobic} / (p_{anaerobic} + 15(1- p_{anaerobic})) \quad (1)$$

The increased glucose turnover in g/kg patient/day is converted to increased mol glucose/kg patient/day using the molecular mass of glucose, 180.16 g/mol, to get mol glucose/kg patient/day increase. Based on $X_{anaerobic}$, the amount of glucose used anaerobically by the tumor is calculated. This leads to mol glucose/kg patient/day recycled in the Cori cycle. 6 mol ATP is needed per mol glucose/kg/day recycled, leading to mol ATP/kg/day Cori cycling cost. Assuming the liver generated its energy aerobically, we use 30 mol ATP/mol glucose consumed by the liver, to get a mol glucose consumed by liver/kg/day Cori cycle cost. We convert this to g glucose consumed by liver/kg/day for Cori cycle, using again 180.16 g/mol molecular weight of glucose. Then we convert this value to kcal/kg/day using the conversion parameter of 4.2 kcal/g used by Hall [1] to get the Cori cycle cost in kcal/kg/day. As seen in Table S1, the value of $X_{anaerobic}$ leads to the percentage the Cori cycle cost is of the total cost of the cancer. The total cost of the cancer is then calculated in kcal/kg patient/day. In Table 2, the cost, $P_{cost\_glucose}$, is then displayed for a 70kg patient.

In study B, where an estimate of the tumor burden is available, we are able to use the same method to calculate the specific cost of cancer, K_{cost}, for a 70kg patient, with the assumption of $X_{anaerobic}$=25%.

References

1. Hall KD: **Computational model of in vivo human energy metabolism during semistarvation and refeeding.** *Am J Physiol Endocrinol Metab* 2006, **291:**E23-E37.
