# Supplementary material for: Modeling the energetic cost of cancer as a result of altered energy metabolism: implications for cachexia
Source: Theor Biol Med Model. 2015 Sep 15;12:17. doi: 10.1186/s12976-015-0015-0 (PMC4570294; doi:10.1186/s12976-015-0015-0)
Supplement: Additional file 5: — Recurrence relation g(p cancer , p anaerobic ). Additional explanation is given to the recurrence relation g(pcancer , p anaerobic ) and how it is used to calculate p lost. (PDF 70 kb) [file 12976_2015_15_MOESM5_ESM.pdf]

Additional file 5: Recurrence relation $g(p_{cancer}, p_{anaerobic})$

$g(p_{cancer}, p_{anaerobic})$, the expected ATP generated for the body per glucose entering the bloodstream (energetic payout of a glucose), is described by a recurrence relation:

$$g_{(t+1)}(p_{cancer}, p_{anaerobic}) = (1- p_{cancer})30 + p_{cancer}(p_{anaerobic}(-6 + g_{(t)}(p_{cancer}, p_{anaerobic}))) \quad (1)$$

This recurrence relation describes roughly what may happen to a glucose in the bloodstream. In the first case (the first term of equation 1), the glucose is consumed by the body, with probability ($1- p_{cancer}$), and generate 30 ATP for the body.
In the second case (the second term of equation 1), the cancer consumes the glucose, with probability p_{cancer}. In this case, with probability $p_{anaerobic}$, the cancer metabolizes the glucose anaerobically, which leads to lactate being produced. For the body, this costs 6 ATP in the Cori cycle to recycle the glucose. Since the glucose is recycled, it leads to the term $g_{(t)}(p_{cancer}, p_{anaerobic}))$ in equation 1 which signifies that $g(p_{cancer}, p_{anaerobic})$ has to be recalculated for that glucose because it is back in the bloodstream at the next time point ($t+1$). If the cancer uses the glucose aerobically, the body does not get any ATP from this glucose, and so its value is 0 to the body, and so it is omitted from equation 1. t in equation 1 signifies time, starting at t=0. Because every time the glucose entering the bloodstream has a chance of being used by the tumor anaerobically and being recycled, it leads to an increase in time t, and the recurrence relation continues. As t increases, there is less and less chance of the original glucose not being consumed either by the body or aerobically by the tumor. $g_{(t)}$ converges to an expected payout of ATP per glucose as t (time) goes to infinity. If no glucose was lost to the cancer, $g_{(t)}$ would be 30 ATP. The initial condition $g_{(0)}$ is given to be 30 ATP, although this does not affect the final solution of $g_{(t)}$ as t goes to infinity. The value of $g(p_{cancer}, p_{anaerobic})$ is the value of $g(p_{cancer}, p_{anaerobic})$ as t goes to infinity.

p_{lost} , the percentage of energy lost to the body per glucose entering the bloodstream, is calculated as follows:

$$p_{lost}(p_{cancer}, p_{anaerobic}) = (30 - g(p_{cancer}, p_{anaerobic})) / 30 \quad (2)$$

Because the tumor consumes glucose, the body will not get the full 30 ATP that would be normally expected in a healthy body. For example, if the probability that glucose enters the cancer is 25%, and the probability that the cancer consumes the glucose anaerobically is 75%, then p_{cancer} is 0.25, $p_{anaerobic}$ is 0.75, g(0.25, 0.75) is 26.3, and so p_{lost} is 12%. In effect, The expected amount of ATP generated for the body in this case is 26.3 ATP, when in a healthy body it would be 30 ATP. So the percentage of energy lost is 12%.
